# Prognosis and Predictors of Symptomatic Intracranial Hemorrhage After Endovascular Treatment of Large Vessel Occlusion Stroke

**DOI:** 10.3389/fneur.2021.730940

**Published:** 2022-01-21

**Authors:** Huixin Shen, Qingfeng Ma, Liqun Jiao, Fei Chen, Sufang Xue, Jingya Li, Zhengping Li, Haiqing Song, Xiaoqin Huang

**Affiliations:** ^1^Departments of Neurology, Xuanwu Hospital, Capital Medical University, Beijing, China; ^2^Departments of Neurosurgery, Xuanwu Hospital, Capital Medical University, Beijing, China

**Keywords:** acute ischemic stroke, large vessel occlusion, endovascular treatment, symptomatic intracranial hemorrhage, predictors

## Abstract

**Background:**

Symptomatic intracranial hemorrhage (sICH) is a devastating complication of endovascular treatment (EVT) in patients with acute ischemic stroke (AIS) and is associated with high risk of disability and mortality. This study intended to evaluate the predictors of sICH after EVT in patients with large vessel occlusion (LVO)-induced AIS.

**Methods:**

We conducted a retrospective review on consecutive AIS patients who underwent EVT in our University hospital between January 2019 and August 2020. The patients were classified into two groups based upon the occurrence of sICH. The main outcomes were the occurrence of sICH using the Heidelberg Bleeding Classification and functional condition at 90 days. Multivariate logistic regression analysis and receiver operating characteristics (ROC) curves were used to identify independent predictors of sICH after EVT.

**Results:**

Three hundred and 69 patients were enrolled in the study, of which 16.8% (*n* = 62) developed sICH. Favorable neurological outcome was lower in patients with sICH than in patients without sICH (6.5 vs. 43.3%; *P* < 0.001), with the overall mortality being 112 (30.4%) at 90 days post- EVT. Results from univariate analysis showed significant differences between the two groups in the prevalence of diabetes, initial Alberta Stroke Program Early CT Score (ASPECTS) score, National Institutes of Health Stroke Scale (NIHSS) score after operation, the levels of fasting blood glucose (FBG), neutrophil to lymphocyte ratio (NLR), platelets (PLT), and thrombin time (TT) at admission. Multivariate logistic regression analysis showed that FBG ≥ 7.54 mmol/L (OR: 2.765; 95% confidence interval [CI]: 1.513–5.054), NLR ≥ 5.48 (OR: 2.711; 95% CI: 1.433–5.128), TT at admission ≥ 16.25 s (OR: 2.022; 95% CI: 1.115–3.667), and NIHSS score within 24 h after the operation ≥ 10 (OR: 3.728; 95% CI: 1.516–9.170) were independent predictors of sICH. The combination of NLR ≥ 5.48, FBG ≥ 7.54 mmol/L, TT at admission ≥ 16.25 s, and NIHSS score within 24 h after the operation ≥ 10 generated an optimal prediction model (AUC: 0.723).

**Conclusion:**

Higher levels of FDG, NLR, TT at admission, and NIHSS score after operation were associated with sICH after EVT in patients with LVO-induced AIS.

## Introduction

Stroke is the second and the third most common cause of death and disability, respectively, globally (1). Acute ischemic stroke (AIS) arises from large vessel occlusion (LVO) has serious effects on the survival and quality of life of patients since it is associated with high risks of disability and mortality. Currently, reperfusion therapies are the most effective therapeutic strategies for AIS (2). Endovascular treatment (EVT) with or without intravenous thrombolysis (IVT) has become the standard of care for AIS caused by anterior circulation large-vessel occlusions based on findings from several randomized controlled trials (3–7).

Despite the success associated with endovascular treatment, complications of EVT such as symptomatic intracranial hemorrhage (sICH) can reduce the benefit-risk ratio of the treatment (8). However, there is limited data available regarding the predictors and clinical relevance of sICH after EVT in patients with LVO-induced AIS. In addition, the reported risk factors vary by country and region. Therefore, it is of great significance to identify the risk factors for sICH after EVT for the prevention of sICH and the improvement of the efficacy of this new treatment strategy.

The propose of our study was to identify the potential predictors of sICH after EVT in individuals with LVO-induced AIS by analyzing clinical data collected from our center. This will help guide the development of appropriate management strategies against the sICH.

## Subjects and Methods

### Patients

We conducted a single-center retrospective cohort study of patients who were treated with EVT for AIS between January 1, 2019, and August 3, 2020, at Xuanwu Hospital of Capital Medical University, China.

Patients were included in the study if (1) patients were diagnosed with AIS; (2) large vessel occlusion (LVO) was identified as the cause of AIS using computed tomography angiography (CTA), brain magnetic resonance angiography (MRA), and/or cerebral digital subtraction angiography (DSA); (3) they received endovascular recanalization therapy (including intra-arterial thrombolysis, mechanical thrombectomy with or without stenting).

Exclusion criteria: patients (1) had no follow-up brain imaging (Computed tomography or Magnetic resonance imaging) at 24 h or when neurological deterioration occurred; (2) only received DSA without further treatment; and (3) had no modified Rankin Scale (mRS) at 3 months.

### Clinical Data Collection

The electronic medical records were reviewed and analyzed for data such as baseline demographic data (age and gender), Body Mass Index (BMI), vascular risk factors (smoking, drinking, hypertension, hyperlipidemia, diabetes, atrial fibrillation, previous stroke, and coronary heart disease), blood pressure at admission (systolic and diastolic), pre-stroke mRS, stroke severity, stroke subtype, radiographic features [Alberta Stroke Program Early CT Score (ASPECTS), the site of the occluded arteries], and information concerning EVT (procedure process time, treatment methods and recanalization). Additional data that was analysed include Fasting blood glucose (FBG), white blood cell (WBC), platelets (PLT), thrombin time (TT), neutrophil to lymphocyte ratio (NLR), albumin, total cholesterol (TC), triglyceride (TG), and low-density lipoprotein (LDL). NLR was defined as the ratio of the absolute neutrophil count to the absolute lymphocyte count.

The stroke severity of the patients at admission was estimated using the National Institutes of Health Stroke Scale (NIHSS). The stroke subtype was classified according to Trial of Org 10,172 in Acute Stroke Treatment (TOAST) criteria (9). The site of the occluded arteries was identified using CTA, MRA, and/or cerebral DSA reports, and included internal carotid artery (ICA), middle cerebral artery (MCA), anterior cerebral artery (ACA), vertebral artery (VA), and basilar artery (BA). Anterior circulation lesions were defined using ASPECTS and posterior circulation lesions were evaluated using pc-ASPECTS.

The procedure process time involved symptom onset-to-door time (OTD), door-to-groin puncture time (DTP), and puncture-to-final recanalization time (PTR). Patients in our study received endovascular treatment, which included intra-arterial thrombolysis, thrombectomy with stent retrievers, thromboaspiration, intracranial angioplasty and stent implantation, or a combination of these approaches at the discretion of the treatment surgeon.

Successful reperfusion was defined as a modified Thrombolysis in Cerebral Infarction (mTICI) score of 2b or 3 (10). The modified Rankin scale (mRS) was conducted at 3 months by a stroke neurologist during a scheduled post-stroke follow-up visit, or *via* a phone interview. Functional outcome was evaluated according to mRS as complete recovery (mRS = 0–1); partial recovery, independent (mRS = 2); dependent (mRS = 3–5); and death (mRS = 6). Favorable and unfavorable outcomes were defined as mRS of 0–2 and > 2 (3–6), respectively.

### Evaluation of sICH

Symptomatic Intracranial Hemorrhage (sICH) was classified based on the Heidelberg Bleeding Classification (11). The diagnosis of sICH was based on the association of ICH with any of the following conditions: (1) Increase in NIHSS score by >4 points compared to the score before ICH; (2) Increase in NIHSS score by >2 points in one category; (3) deterioration leading to intubation, hemicraniectomy, external ventricular drain placement, or any other major interventions. It is also necessary that the symptom deteriorations cannot be explained by causes other than the observed ICH (11). For hemorrhage classified as parenchymal hematoma 2 (PH 2, hematoma occupied ≥ 30% of the infarct volume, with an obvious mass effect), even if the neurological function deteriorations could be attributed to infarction, the hemorrhage would be defined as sICH.

### Statistical Analysis

All statistical analyses were performed using SPSS Statistics 25.0 software (IBM, Armonk, NY, USA). Baseline characteristics were compared between patients with sICH and patients without sICH. Continuous variables are shown as medians (interquartile ranges, IQRs) according to the type of non-normal distribution, while categorical variables are presented as frequencies (percentages). Mann-Whitney *U* tests were used for continuous variables, and χ^2^ tests or Fisher exact tests were adopted for categorical variables. For the functional outcome, an ordinal logistic regression analysis was performed to evaluate the effect of sICH across the entire range of mRS scores. Then, we used the binary logistic regression analysis to evaluate independent predictors for sICH, with the adjusted ORs and corresponding 95% CIs being reported. Receiver operating characteristic (ROC) curve analysis was used to evaluate the optimal cutoff value for predicting sICH and to establish optimal cutoff points at which the sum of the specificity and sensitivity was the highest.

Results of univariate analyses with *P* < 0.1 were involved in the multivariate logistic regression. The overall ROC analysis was used to valuate the overall discriminative ability of the five-items model to predict sICH after EVT. A two-tailed value of *P* < 0.05 was considered to be significant.

## Results

### Clinical Characteristics of the Patients

**Figure 1:**
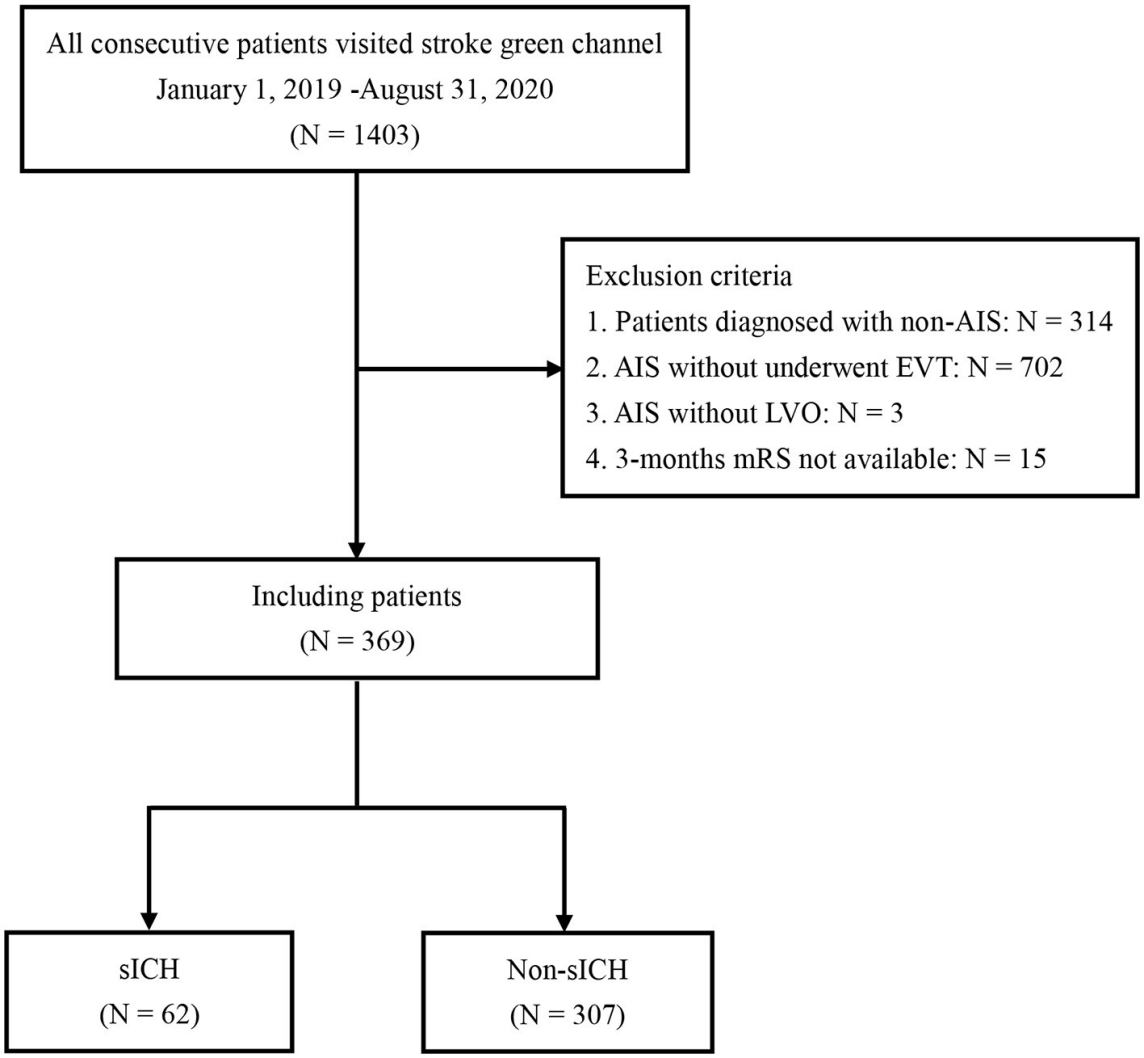
Flowchart of patient selection. AIS, acute ischemic stroke; LVO, large vessel occlusion; EVT, endovascular treatment; sICH, symptomatic intracranial hemorrhage.

Out of the 1,403 patients who visited the emergency department at Xuanwu Hospital during the study period, we excluded 1,034 patients based on the inclusion and exclusion criteria. A total of 369 patients with AIS due to large vessel occlusion treated with EVT and were enrolled in this study. A study population flowchart is shown in Figure 1.

Among the 369 patients, the median age for the cohort was 66 (57, 74) years, and 67.8% were male. The median baseline NIHSS and ASPECTS scores were 15 (12, 19) and 8 (7, 9), respectively. According to the TOAST classification, 257 patients were defined as large-artery atherothrombotic (69.6%), 95 as cardioembolic (25.7%), and 17 (4.6%) as other subtypes of stroke.

The sites of artery occlusion included ICA (118, 32%), MCA (156, 29.5%), ICA + MCA (2, 0.5%), VA (18, 4.9%), and BA (75, 20.3%). Occlusion of the anterior circulation was recorded in 74.8% (276/369) of the patients. The median time from symptom OTD, DTP, OTP, and PTR were 233 (127, 344) min, 130 (105, 162) min, 366 (279, 492) min, and 40 (29, 58) min, respectively.

Thrombectomy with stent retrievers and aspiration (a) was performed in 256 patients (69.4%), intracranial angioplasty and stent implantation (b) in 22 patients (6.0%), and a combination of a and b in 78 patients (21.1%). In addition, 11 patients (3%) were treated with intra-arterial thrombolysis, whereas two patients (0.5%) were treated with both intra-arterial thrombolysis and stent implantation. A total of 80 patients (21.7%) underwent IVT before EVT. Successful reperfusion was reported in 87.3% of the patients and the median NIHSS score within 24 h after EVT was 13 (8, 19). Demographic, clinical, and Laboratory data are shown in Table 1, while radiological characteristics, procedure process time, and therapy are shown in Table 2.

**Table 1:** Baseline characteristics of patients with sICH and without sICH.

**No**.	**Total Patients (*n* = 369)**	**sICH group (*n* = 62)**	**Non-sICH group (*n* = 307)**	***P*-value**
Age (y)	66 (57, 74)	68 (59, 75)	65 (57, 74)	0.224
Gender, male, *n* (%)	250 (67.8)	38 (61.3)	212 (69.1)	0.233
BMI (kg/m^2^)	24.91 (22.86, 27.34)	24.75 (22.05, 27.19)	24.97 (23.03, 27.34)	0.347
Medical history, *n* (%)				
Smoking	115 (31.2)	16 (25.8)	99 (32.2)	0.318
Drinking	102 (27.6)	14 (22.6)	88 (28.7)	0.329
Hypertension	252 (68.3)	42 (67.7)	210 (68.4)	0.919
Hyperlipemia	95 (25.7)	13 (21.0)	82 (26.7)	0.346
Diabetes	108 (29.3)	28 (45.2)	80 (26.1)	0.003
Atrial fibrillation	97 (26.3)	21 (33.9)	76 (24.8)	0.137
Previous stroke	109 (29.5)	21 (33.9)	88 (28.7)	0.412
Coronary heart disease	89 (24.1)	17 (27.4)	72 (23.5)	0.505
**Blood pressure at admission (mmHg)**				
SBP	150 (135, 167)	159 (136, 171)	150 (135, 165)	0.182
DBP	85 (78, 92)	86 (80, 93)	84 (78, 92)	0.525
**Related scores**				
Pre-stroke mRS score	0 (0, 0)	0 (0, 0)	0 (0, 0)	0.195
ASPECTS score at admission	8 (7, 9)	8 (7, 9)	8 (7, 10)	0.008
NIHSS score at admission	15 (12, 19)	16 (12, 20)	15 (12, 19)	0.198
**Laboratory data**				
FBG (mmol/L)	7.2 (6.0, 9.3)	8.1 (7.0, 12.7)	7.1 (5.9, 9.0)	<0.001
WBC (× 10^9^/L)	8.9 (7.1, 11.0)	9.0 (6.8, 11.3)	9.2 (7.3, 11.1)	0.973
NLR	5.95 (3.38, 9.68)	6.48 (4.02, 9.36)	5.80 (3.08, 10.08)	0.038
PLT (× 10^9^/L)	212 (172, 252)	199 (159, 246)	223 (181, 264)	0.033
TT (s)	16.0 (15.3, 17.0)	16.4 (15.4, 17.2)	15.9 (15.2, 16.5)	0.013
Albumin (g/L)	40.3 (38.0, 42.7)	40.8 (39.1, 42.9)	40.8 (38.5, 43.1)	0.458
TC (mmol/L)	4.44 (3.69, 5.09)	4.61 (3.83, 5.27)	4.43 (3.65, 5.06)	0.740
TG (mmol/L)	1.19 (0.79, 1.81)	1.18 (0.73, 2.05)	1.19 (0.80, 1.83)	0.934
LDL (mmol/L)	2.78 (2.07, 3.46)	2.94 (2.22, 3.67)	2.77 (2.07, 3.46)	0.799
Stroke subtype: TOAST, *n* (%)				0.411
LAA	257 (69.6)	40 (64.5)	217 (70.7)	
CE	95 (25.7)	20 (32.3)	75 (24.4)	
Other subtype	17 (4.6)	2 (3.2)	15 (4.9)	

*sICH, symptomatic intracranial hemorrhage; BMI, Body Mass Index; SBP, Systolic blood pressure; DBP, Diastolic blood pressure; mRS, modified Rankin scale; APSECTS, Alberta Stroke Program Early Computed Tomography score; NIHSS, National Institutes of Health Stroke Scale; FBG, Fasting blood glucose; WBC, White blood cell; NLR, Neutrophil to lymphocyte ratio; PLT, Platelets; TT, Thrombin time; TC, Total cholesterol; TG, Triglyceride; LDL, Low-density lipoprotein; TOAST: Trial of Org 10172 in Acute Stroke Treatment; LAA, Large-artery atherosclerosis; CE, Cardioembolism*.

**Table 2:** Procedural and treatment of patients with sICH and without sICH.

**No**.	**Total Patients (*n* = 369)**	**sICH group (*n* = 62)**	**Non-sICH group (*n* = 307)**	***P*-value**
Site of artery occlusion, *n* (%)				0.665
ICA	118 (32)	23 (37.1)	95 (30.9)	
MCA	156 (29.5)	28 (45.2)	128 (41.7)	
ICA + MCA	2 (0.5)	0 (0.0)	2 (0.7)	
VA	18 (4.9)	2 (3.2)	16 (5.2)	
BA	75 (20.3)	9 (14.5)	66 (21.5)	
Lesion site, *n* (%)				0.138
Anterior circulation lesions	276 (74.8)	51 (82.3)	225 (73.3)	
Posterior circulation lesions	93 (25.2)	11 (17.7)	82 (26.7)	
Procedure process time (min)				
OTD	233 (127, 344)	220 (120, 347)	229 (127, 334)	0.982
DTP	130 (105, 162)	125 (99, 182)	130 (107, 161)	0.826
OTP	366 (279, 492)	366 (283, 469)	369 (274, 493)	0.951
PTR	40 (29, 58)	39 (27, 54)	40 (29, 58)	0.638
Therapy, *n* (%)				0.957
Thrombectomy with stent retrievers + aspiration (a)	256 (69.4)	45 (72.6)	211 (68.7)	
Intracranial angioplasty + stent implantation (b)	22 (6.0)	3 (4.8)	19 (6.2)	
a + b	78 (21.1)	12 (19.4)	66 (21.5)	
Intra-arterial thrombolysis	11 (3.0)	2 (3.2)	9 (2.9)	
Intra-arterial thrombolysis + stent implantation	2 (0.5)	0 (0.0)	2 (0.7)	
Intra-arterial tirofiban, *n* (%)	92 (24.9)	11 (18.0)	81 (26.4)	0.169
IVT + EVT, *n* (%)	80 (21.7)	13 (21)	67 (21.8)	0.881
mTICI at end of procedure, *n* (%)				0.966
0-2a	47 (12.7)	9 (6.6)	38 (16.4)	
2b-3	322 (87.3)	54 (87.1)	268 (87.3)	
NIHSS score after operation	13 (8, 19)	16 (12, 25)	13 (8, 18)	<0.001

*sICH, symptomatic intracranial hemorrhage; ICA, intracranial carotid artery; MCA, middle cerebral artery; VA, Vertebral artery; BA, Basilar artery; OTD, symptom onset to door time; DTP, door to groin puncture time; OTP, symptom onset to groin puncture time; PTR, puncture to final recanalization time; MT, mechanical thrombectomy; IVT, intravenous thrombolysis; EVT, endovascular treatment; mTICI, Modified Treatment in Cerebral Ischemia Scale; NIHSS, National Institutes of Health Stroke Scale*.

### Correlation Between sICH and mRS at 90 Days

**Figure 2:**
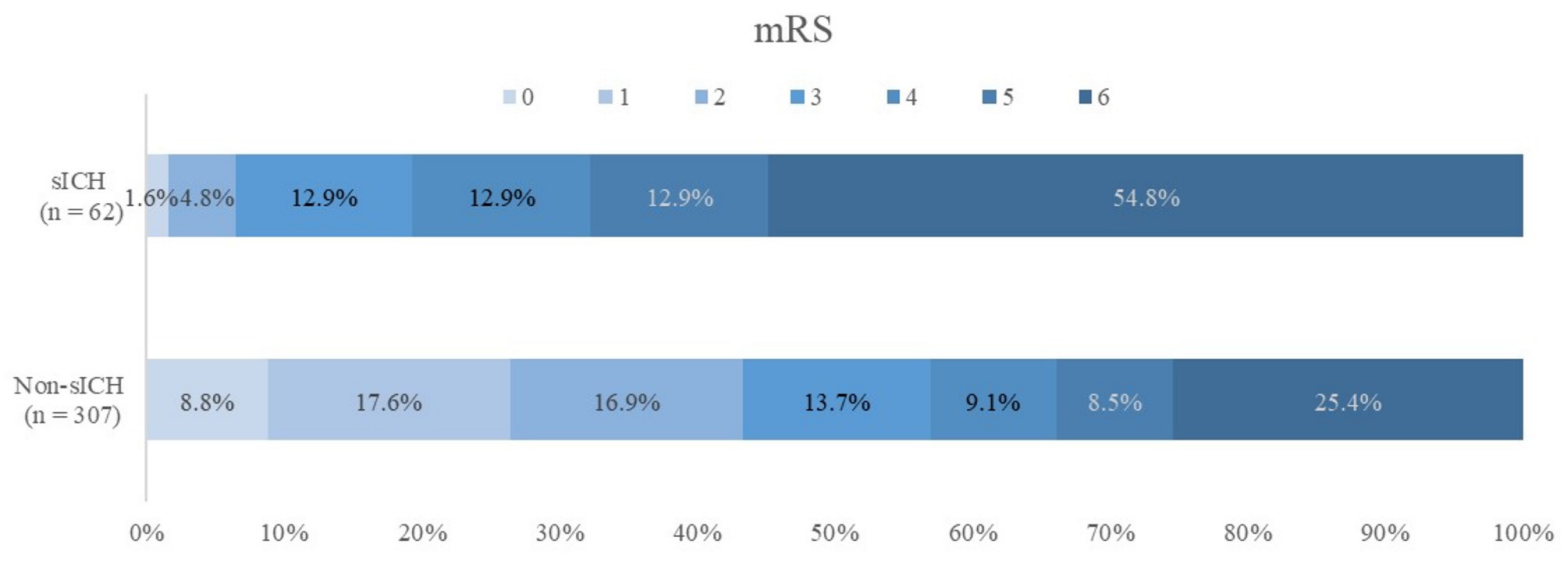
Modified Rankin Scale (mRS) scores at 90 d in patients with and without symptomatic intracranial hemorrhage (sICH). There was a statistically significant difference between two groups in the overall distribution of mRS scores in an analysis with univariable ordinal regression (common odds ratio, 0.23; 95% confidence interval, 0.957–2.020), indicating a shift toward poor functional outcome, with non-sICH patients as reference group.

The patients with sICH who reached a favorable functional outcome (mRS, 0–2) at 90 days were less than patients without sICH who had favorable functional outcome (6.5 vs. 43.3%; *P* < 0.001). The overall mortality was 112 (30.4%) at 90 days after EVT, with the mortality being higher for patients with sICH (54.8 vs. 25.4%, *P* < 0.001) (Table 3). The overall distribution of 90-day mRS scores in the sICH and non-sICH groups is shown in Figure 2.

**Table 3:** Functional outcome at 90 days in sICH and non-sICH groups.

	**Total**	**sICH group (*n* = 62)**	**Non-sICH group (*n* = 307)**	***P*-value**
mRS at 90 d				<0.001
mRS 0–2	137 (37.1)	4 (6.5)	133 (43.3)	
mRS 3–6	232 (62.9)	58 (93.5)	174 (56.7)	
mortality	112 (30.4)	34 (54.8)	78 (25.4)	<0.001

*sICH, symptomatic intracranial hemorrhage; mRS, modified Rankin Scale*.

### Comparison Between Patients With and Without sICH

Sixty-two patients (16.8%) were diagnosed with sICH within 72 h after EVT. The characteristics of patients with sICH are shown in Tables 1, 2.

In the univariate analysis, the prevalence of diabetes was higher in sICH group than in non-sICH group (45.2 vs. 26.1%, *P* = 0.003). The initial ASPECTS scores after EVT were lower in patients with sICH compared to the patients without sICH [8 (7, 9) vs. 8 (7, 10), *P* = 0.008]. In addition, there were significant differences in FBG (*P* < 0.001), NLR (*P* = 0.038), PLT (*P* = 0.033), and TT at admission (*P* = 0.013) between the two groups of patients. However, there were no significant differences in WBC counts, albumin, TC, TG, or LDL (*P* > 0.05) at admission between the two groups. The NIHSS score within 24 h after EVT was higher in the patients with sICH than in the patients without sICH [16 (12, 25) vs. 13 (8, 18), *P* < 0.001].

Univariate logistic regression analysis revealed that ASPECTS score at admission, FBG, NLR, PLT, TT, and NIHSS score after the operation were associated with sICH after EVT. ROC analyses were performed to identify the optimal cutoff values of ASPECTS score at admission, FBG, NLR, PLT, TT, and NIHSS score after operation for predicting sICH. The optimal cutoff value for ASPECTS scores at admission FBG, NLR, PLT, TT, and NIHSS score after the operation was ≤ 6, ≥ 7.54 mmol/L, ≥ 5.48, ≤ 478 × 10^9^/L, ≥ 16.25 s, and ≥ 10, respectively.

After adjusting for all potential confounders, FBG ≥ 7.54 mmol/L [adjusted odds ratio (OR): 2.765; 95% confidence interval (CI): 1.513–5.054], NLR ≥ 5.48 (OR: 2.711; 95% CI: 1.433–5.128), TT at admission ≥ 16.25 s (OR: 2.022; 95% CI: 1.115–3.667), and NIHSS score after operation ≥ 10 (OR: 3.728; 95% CI: 1.516–9.170) were identified as independent predictors for sICH after EVT (*P* < 0.05). Table 4 shows the results of the multivariate logistic regression model used to determine predictors of sICH after EVT.

**Table 4:** Multivariate analysis of predictors of sICH after EVT.

**Characteristics**	**OR**	**95% CI**	***P*-value**
ASPECTS score at admission ≤ 6	1.021	0.332–3.144	0.971
FBG ≥ 7.54 mmol/L	2.765	1.513–5.054	0.001
NLR ≥ 5.48	2.711	1.433–5.128	0.002
PLT ≤ 478 × 10^9^/L	2.739	0.196–38.235	0.454
TT ≥ 16.25 s	2.022	1.115–3.667	0.021
NIHSS score after operation ≥ 10	3.728	1.516–9.170	0.004

*sICH, symptomatic intracranial hemorrhage; EVT, endovascular treatment; OR, Odds ratio; 95% CI, 95% confidence interval; APSECTS, Alberta Stroke Program Early Computed Tomography score; FBG, Fasting blood glucose; NLR, Neutrophil to lymphocyte ratio; PLT, Platelets; TT, Thrombin time; NIHSS, National Institutes of Health Stroke Scale*.

**Figure 3:**
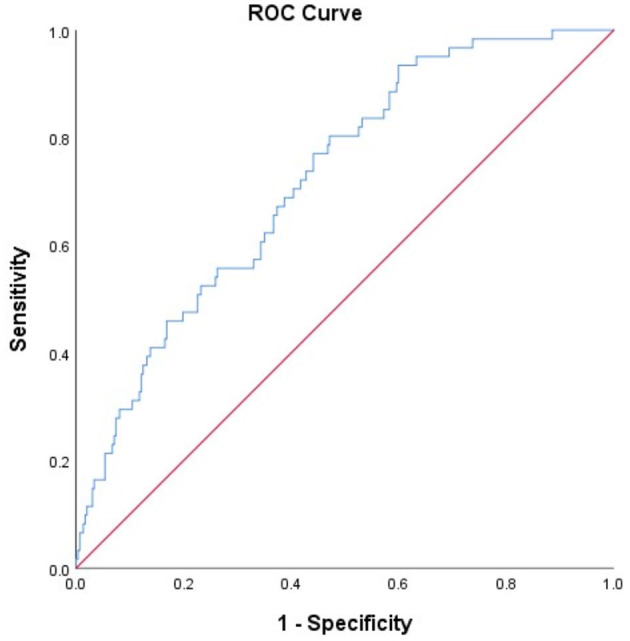
Receiver operating characteristic (ROC) curve for the value of model to predict sICH. The ROC curves showing the predictive ability of the model (including ASPECTS score at admission, NIHSS score after operation, FBG, NLR, PLT, and TT levels). Area under the curve of the model: 0.723.

**Figure 4:**
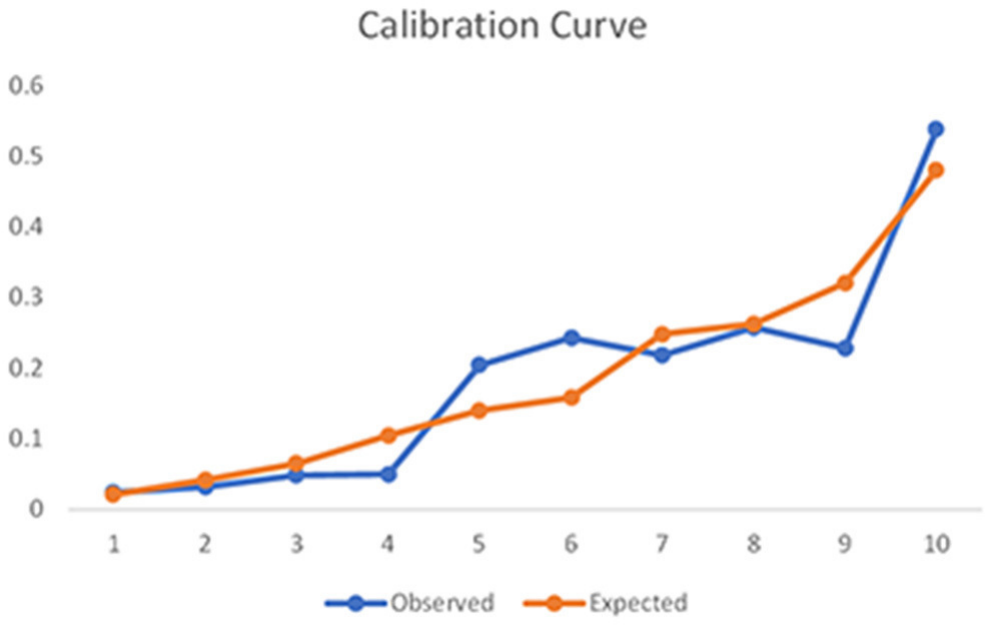
Calibration curve for the model to predict sICH. The calibration curve for the model showing the calibration ability of the model. Hosmer-Lemeshow χ^2^ = 6.924, *P* = 0.549 > 0.05.

Figure 3 shows the results of ROC analysis for determining the prognostic value of the model to predict sICH. The area under the curve for the model was 0.723, which indicates that the model has a good overall discriminative ability. Figure 4 shows the calibration curve for the model to predict sICH. The calibration curve for the model showing the calibration ability of the model. Hosmer-Lemeshow χ^2^ = 6.924, *P* = 0.549 > 0.05.

## Discussion

In our study, we explored the predictors of sICH after EVT in patients with large vessel occlusion (LVO)-induced AIS. We demonstrated that higher levels of FDG, NLR, TT at admission, and NIHSS score after operation were associated with sICH after EVT in LVO-induced AIS patients.

In our study, the occurrence of sICH was higher (16.8%) than that reported in randomized controlled trial studies (4.4%) (12) and the North American Solitaire Acute Stroke study (9.9%) (13), but was similar to the ACTUAL study (16%) in China (14). Previous studies (14) have indicated that a longer time from symptoms onset to puncture might be associated with sICH, while in our study no differences were found between procedure process time and type of treatment. This was possibly due to the retrospective nature of our study which better reflects the realities in clinical practice such as expanded indications, unfavorable treatment conditions, or insufficient practices that may result in a higher risk of sICH. In addition, the study was conducted in a national referral center with grievous cases of cerebral vascular disease, which could have introduced a selection bias that did not fully represent the actual conditions of AIS patients after EVT. Similar to reports from previous studies, sICH after EVT was associated with poor functional outcomes and mortality (8). Findings from our study revealed that patients with sICH showed poorer functional outcomes (93.5% of cases) compared to patients without sICH, with a mortality of 54.8%. This was an indication that sICH is a fatal complication that greatly affects the prognosis of patients and should be taken seriously.

In our study, high levels of FBG (≥ 7.54 mmol/L) were the strongest predictor of sICH after EVT. FBG scores higher than 7.54 mmol/L in patients with LVO-induced AIS were associated with increased risk of sICH after EVT, which was consistent with previous studies (15–19). It is well known that hyperglycemia often coexists with AIS and is associated with poor functional outcomes (15). High levels of blood glucose have been reported as a related factors of sICH in patients undergone intravenous thrombolysis (IVT) (16) or intra-arterial thrombolysis (17). A study indicated that patients with a serum glucose level ≥of 160 mg/dl had an extremely higher risk of sICH compared to patients with normal blood glucose levels (18). Furthermore, another study revealed that postoperative glucose values were independent predictors of sICH in patients with anterior circulation LVO treated with stenting. The addition of postoperative glucose values to the basic risk factors model could improve the risk prediction for sICH after EVT (19). Leon et al. observed a linear relationship and an overall significant association between glucose and the probability of sICH and poor functional outcome for patients with glucose levels between 6 mmol/L and 9 mmol/L on admission (20). Several mechanisms underlying the relationship between ICH and hyperglycemia have been proposed. Hyperglycemia has been shown to increase the activity of matrix metalloproteinase (MMP)-9 and MMP-3 in the ischemic region (21), exacerbate blood-brain barrier dysfunction and hemorrhagic transformation after recanalization (22).

Our results also shown that high NLR is a strong predictor of sICH after EVT. The study of Lee et al. (23) and Forget et al. (24) reported a mean NLR of 1.65 and a median NLR of 1.65 in the adult healthy population, respectively. In a previous cohort of 143 AIS patients with LVO in the anterior circulation, NLR measured at admission predicted ICH after endovascular thrombectomy, with a cut-off value of 3.89 (25). Hence, our study expands the existing literature by analyzed the relationship between NLR on admission and sICH after EVT in patients with LVO-induced AIS. Our study identified a cut-off of five. Forty-eight (72.1% sensitivity and 50.5% specificity), and a median NLR of 6.48 for the sICH group, which is much higher than that in the healthy individuals. NLR refers to the Neutrophil-to-Lymphocyte Ratio and has gained interest in the recent years as a biomarker of inflammation. Previous studies have indicated that NLR is a predictor for unfavorable functional outcome after AIS and ICH, but the underlying mechanisms remain indefinite (26–28). The occurrence of AIS results in an increase in neutrophil counts (an activation of neutrophils), and a decrease in lymphocyte counts due to the post-stroke immunodepression (29), activated by the sympathetic nervous system and hypothalamic-pituitary-adrenal axis (30). The subsequent increase in the secretion of MMP-9 causes disruption of the neurovascular unit, which explains the occurrence of ICH in AIS patients with higher NLR. The role of neutrophils is even more complex as pro-inflammatory N1 neutrophils are involved in brain neurotoxicity, whereas anti-inflammatory N2 neutrophils have been found to prompt neuronal survival and successful brain reconstruction (31).

In our study, we observed that TT at admission predicted sICH after EVT, with a cut-off value of 16.25s. As far as we know, our research is the first study to evaluate the relationship between TT at admission and sICH in AIS patients after EVT. We therefore made inferences using findings from studies on the relationship between cerebral hemorrhage and coagulation. TT is prolonged with low levels of fibrinogen, dysfunctional fibrinogen, or when inhibitors of thrombin are present, indicating a decrease in coagulation function. A study found that coagulation function was associated with cerebral microbleeds (CMBs) and confirmed that activated partial thromboplastin time (APTT) was an independent predictor for CMBs (32). In addition, low circulating fibrinogen levels were associated with post-thrombolysis hemorrhage in patients with AIS (33). Zsuzsa Bagoly et al. investigated the thrombin generation test in AIS patients before IVT and found that sICH was significantly correlated with low endogenous thrombin potential (ETP) and thrombin peak levels (33). Therefore, we speculated that the thrombin generation test may be a useful tool for predicting outcomes and safety of recanalization therapy in prospective studies.

The conclusions regarding the relationship between NIHSS and ICH are inconclusive due to inconsistent results reported from different studies. Some research shown that the baseline NIHSS score was independently associated with sICH (34, 35), While another IVT study showed that baseline NIHSS score was not independently associated with sICH after adjustment for the aforementioned covariates (36). In contrast to previous studies, we evaluated the NIHSS score at admission and within 24 h after EVT, and found that only postoperative NIHSS is statistically significant. This may indicate that the higher NIHSS score within 24 h after EVT may be related to ischemic cerebral hemorrhage in clinical practice. In the future, further researches are still needed to verify.

There were some potential limitations to our study due to its retrospective design and use of data from a single-center. In addition, the relatively small sample size and incomplete evaluation index may not fully determine a causal connection. On the other hand, differences in genetic background and design may have contributed to the inconsistencies observed between our results and findings from published studies. The practical implications of these unsolved findings will need future validation. Despite these limitations, the findings from this study give insight into the influence of laboratory results on ICH and prognosis before thrombectomy, to facilitate early management.

## Conclusion

In this single-center study, the incidence of sICH after EVT is higher than previously reported. Incidence of unfavorable functional outcome and mortality is higher for AIS patients with sICH after EVT than those without sICH. High levels of FDG, NLR, TT at admission, and NIHSS score after operation may increase the risk of sICH after EVT in patients with LVO-induced AIS.

## Data Availability Statement

The raw data supporting the conclusions of this article will be made available by the authors, without undue reservation.

## Ethics Statement

The studies involving human participants were reviewed and approved by the Ethics Committee of Xuanwu Hospital, Capital Medical University. The patients/participants provided their written informed consent to participate in this study.

## Author Contributions

XH and HSo responsible for research conception and methodological design, wrote the original manuscript, and prepared to review and editing. QM, LJ, FC, SX, JL, and ZL assisted with participant recruitment and data entry. HSh assisted with data analysis and interpretation. All authors have read and approved the final version of the manuscript.

## Funding

The project was funded by the National Key Research and Development Program of China (2016YFC1300600 and 2016YFC0901004).

## Conflict of Interest

The authors declare that the research was conducted in the absence of any commercial or financial relationships that could be construed as a potential conflict of interest.

## Publisher's Note

All claims expressed in this article are solely those of the authors and do not necessarily represent those of their affiliated organizations, or those of the publisher, the editors and the reviewers. Any product that may be evaluated in this article, or claim that may be made by its manufacturer, is not guaranteed or endorsed by the publisher.
